# Rosuvastatin and chronic hepatitis C

**Published:** 2011-05-01

**Authors:** Eric M. Yoshida

**Affiliations:** 1 Division of Gastroenterology, University of British Columbia, Vancouver BC, Canada

**Keywords:** Rosuvastatin, Chronic hepatitis C, Interferon, Ribavirin

Dear Editor,

The prospective randomized clinical trial of Malaguarnera and colleagues, published in the current issue of Hepatitis Monthly, investigates the potential role of a commercially available HMG Co-A reductase agent-rosuvastatin (Crestor, Astra Zeneca)-in combination with non-pegylated interferon and ribavirin in the treatment of chronic hepatitis C (HCV). HMG Co-A reductase agents, commonly referred to as statins, are popular agents prescribed throughout the world, for their cholesterol lowering effects in order to reduce the risk of cardiovascular morbidity and mortality. They are well-recognized to improve liver biochemistry in dyslipidemic patients with non-alcoholic fatty liver disease [1]; but recent reports have suggested that they may possess an antiviral effect on HCV independent of their lipid lowering activity. In an in vitro study [2], various statin agents were reported to have differing effects on HCV replication in combination with interferon with fluvastatin (Lescol, Novartis) exhibiting the strongest anti-HCV activity, atorvastatin (Lipitor, Pfizer) had moderate activity whereas pravastatin (Pravachol, Bristol Myers Squibb) had no activity. Likewise, the combination of statins-specifically simvistatin and mevastatin-in combination with protease/polymerase inhibitor agents were found to clear HCV replicons from culture [3]. It is interesting that this in vitro study also found that pravastatin exhibited no antiviral activity [3]. Clinically, the experience of statin agents in the treatment of HCV has not been very well-studied and the reported outcomes have been interesting yet at times conflicting. Fluvastatin monotherapy was reported to produce a modest 1.75 log decrease in HCV viral load in HCV monoinfected patients [4]. The same statin, however, resulted in a paradoxical increase in viral load in a study of 42 HIV-HCV co-infected patients [5], presumably, as a result of a statin-dependent up-regulation of low density lipoprotein (LDL) cholesterol receptors that are also required for HCV entry into cells. In combination with peginterferon and ribavirin therapy, fluvastatin has been reported to be associated with an increased likelihood of rapid virologic response (RVR) after 4 weeks if not a sustained virologic response in HIV-HCV co-infected patients [6] and a small open label single arm study of high viral load HCV monoinfected genotype 1 patients reported a suggestion of an enhanced SVR [7].

Given the lack of published randomized clinical trials in this area, the paper by Malaguarnera et al. is therefore of interest. These investigators from the University of Catania, Italy, randomized 65 HCV-infected patients, the overwhelming majority of whom had genotype 1 infection, to receive either rosuvastatin 5 mg/day or placebo, in combination with non-pegylated interferon and ribavirin. Improvements were noted in liver biochemistry, lipid profile, markers of insulin resistance and histology (steatosis and fibrosis) favoring the rosuvastatin arm. Of interest is the reported statistically significant advantage in SVR favoring the rosuvastatin arm: an apparent overall SVR of 69% vs. 62% in the cohort of presumably treatment naive and relapse patients and 51% vs. 40% in presumably treatment naive patients. Although these results appear to be impressive, restraint must be advised when interpreting this study. The absolute numbers of patients are small as the absolute difference in SVR between the two study arms was three patients overall and four patients in the treatment naive group. Moreover, the reported SVR with non-pegylated interferon and ribavirin is much higher than that one would expect from the registration trial of peginterferon vs. non-peginterferon and ribavirin [8]. One would also have to wonder what potential role statin agents would have in the upcoming era of protease inhibitors that promise an SVR of 60%-75% in combination with peginterferon and ribavirin [9][10]. Nevertheless, the work of Malaguarnera and colleagues is certainly hypothesis generating and given that the protease inhibitors are specific for genotype 1 patients, statin agents, that appear to be non-toxic in HCV patients, may have an adjuvant role in non-genotype 1 patients for whom no direct acting antiviral agents (DAA) are on the horizon. Considering that the previously mentioned in vitro study suggests a role in enhancing the effect of protease and polymerase inhibitors [3], it is conceivable that adding a statin to the triple combination of protease/polymerase inhibitor, peginterferon and ribavirin may improve on the impressive SVRs of these DAA medications. Clearly, more clinical trials are needed with these agents.
